# The diagnostic value of the ultrasound gray scale ratio for different sizes of thyroid nodules

**DOI:** 10.1002/cam4.2653

**Published:** 2019-11-05

**Authors:** Xiaoyu Chen, Ming Gao, Linfei Hu, Jialin Zhu, Sheng Zhang, Xi Wei

**Affiliations:** ^1^ Department of Diagnostic and Therapeutic Ultrasonography Key Laboratory of Cancer Prevention and Therapy Tianjin Medical University Cancer Institute and Hospital National Clinical Research Center for Cancer Tianjin's Clinical Research Center for Cancer Tianjin China; ^2^ Department of Thyroid and Cervical Tumor Key Laboratory of Cancer Prevention and Therapy Tianjin Medical University Cancer Institute and Hospital National Clinical Research Center of Cancer Tianjin China

**Keywords:** echogenicity, nodular goiters, papillary thyroid carcinoma, ultrasonic diagnosis, ultrasound gray scale ratio

## Abstract

At present, hypoechogenicity, as one of the clinically relevant features associated with suspicion of malignant thyroid disease, is affected by the variability of modules and the experience of sonographers, thus leading to unsatisfying results. We propose the ultrasound gray scale ratio (UGSR) to obtain an objective, numerical estimate of the echogenicity degree in different‐sized thyroid nodules, and we then evaluate its diagnostic efficacy in differentiating benign and malignant thyroid lesions. In total, 553 ultrasound images of thyroid nodules from one kind of ultrasonographic scanner were analyzed, among which 281 were papillary thyroid carcinomas (PTCs) and 272 were nodular goiters (NGs). The UGSR of the PTCs, NGs, and surrounding normal thyroid tissue was measured by image analysis software. The best cut‐off value for distinguishing various sizes of PTCs and NGs was determined by receiver operating characteristic (ROC) curve analysis. As the UGSR increased, the sensitivity of the diagnosing PTCs decreased, and the specificity increased. When the maximum Jordan index was 0.611, the best cut‐off value was 0.692, and the corresponding sensitivity and specificity of diagnosing PTCs were 87.9% and 73.2%, respectively. For the analysis of subgroups of different tumor sizes, as the size of thyroid nodules increased from 0.3 to 2 cm, the sensitivity of the diagnosis of PTCs decreased from 97.5% to 58.8%, and the specificity increased from 72.4% to 90.9%. These results strongly suggest that the UGSR is an appropriate objective, numerical method for estimating the echogenicity degree and has various diagnostic efficacies in different‐sized thyroid nodules. Thus, the UGSR can be used as an additional ultrasound parameter in the diagnosis of different‐sized PTCs and NGs.

## Graphical abstract

This study investigated the role of ultrasound gray scale ratio (UGSR) for the differentiated diagnosis of malignant and benign thyroid nodules. Using the UGSR to assess the echo was obviously more objective and accurate than the echogenicity identified by the naked eyes. The UGSR can be used as an additional ultrasound parameter in the diagnosis of different‐sized thyroid nodules.

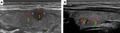

## 1 INTRODUCTION

A steady increase in the incidence of thyroid nodules has been reported due to the widespread use of sensitive imaging methods for screening, which causes overdiagnosis and overtreatment in this setting.^1^, ^2^ Therefore, it is essential to identify as many malignant nodules as possible while excluding those that are highly likely to be benign from fine needle aspiration (FNA) biopsies or surgeries. To achieve precise treatment and to avoid resource waste, many professional institutions, such as the American College of Radiology (ACR) Thyroid Imaging, Reporting and Data System (TI‐RADS),^3^ European TI‐RADS,^4^ and American Thyroid Association guidelines,^5^ have proposed ultrasound‐based risk stratification systems to identify thyroid nodules that warrant biopsy or sonographic follow‐up. Solid composition, hypoechogenicity, taller‐than‐wide shape, irregular margins, extrathyroidal extension, microcalcification, and punctate echogenic foci are clinically relevant features associated with suspicion of malignant disease in these risk stratification systems.^3^, ^6^ Among these features, the echogenicity can be easily affected by the operator's subjectivity compared with ultrasound features such as the morphology, microcalcification, and diameter ratio. A thyroid nodules' echogenicity is related to its composition and cellular structure,^7^ which is a commonly used parameter in ultrasonic examinations. Based on ultrasound images, the echogenicity of thyroid lesions is often divided into five grades (from low to high): nonechoic, extremely hypoechoic (lower than the neck strap muscle echo), hypoechoic (between the neck strap muscle and thyroid echo), iso‐echoic (consistent with the thyroid echo), and hyperechoic (higher than the thyroid echo).^8^, ^9^, ^10^ However, neither the AIUM's guidelines^11^ nor a recent multidisciplinary consensus statement^12^ describes how to assess the echogenicity within muscles or surrounding thyroid tissues. At present, as in the definition of the echogenicity intensity of thyroid nodules, sonographers always depend on subjective, naked‐eye judgment, which is affected by the variability of modules and the experience of sonographers, thus leading to unsatisfying results.

There is a strong correlation between the echogenicity intensity and the gray scale of the ultrasound image. The intensity of the echogenicity on images is shown in black‐to‐white gray scale, which reflects differences between nodules and surrounding tissues. However, the gray scale is also affected by other factors, such as gain level, dynamic range, and frequency. Therefore, there is no applicable method for the gray scale level measurement of all ultrasonic images. However, no matter how the gray scale level changes in thyroid nodules, there is still a “low” and “high” relative ratio between gray scale levels of nodules and the surrounding thyroid tissue. The echogenic appearance of the thyroid gland varies with the adjustment of various instrument settings (gain, depth range, and dynamic range). To meet the need for standard operating conditions, echogenicity is expressed as a ratio in our study. The ultrasound gray scale ratio (UGSR) is defined as the ratio of the gray scale of the thyroid nodules to the surrounding normal thyroid tissues under the same operating conditions. We focus on how to translate the echogenicity information of each nodule into numerical data. The UGSR is used as an available measurement to define a nodule's echogenicity to identify malignant lesions in this study. We used the UGSR to obtain an objective, numerical estimate of the echogenicity degree in different‐sized thyroid nodules and evaluated its diagnostic value in distinguishing benign and malignant thyroid nodules.

## 2 MATERIALS AND METHODS

### 2.1 Study population

This retrospective study was approved by the Ethics Committee of Tianjin Medical University Cancer Institute and Hospital. It required approval from patients for the review of their ultrasound images and records. From October 2013 to September 2016, 1769 thyroid lesions confirmed by surgery and pathology in Tianjin Medical University Cancer Institute and Hospital were divided into the papillary thyroid carcinoma (PTC) group (869 cases) and the nodular goiter (NG) group (900 cases). Nodules with diameters larger than 2.0 cm were excluded due to the lack of surrounding normal thyroid tissues for comparison. Nodules with diameters less than 0.3 cm were excluded to avoid implications of the volume effect on the measurements. Additional exclusion criteria were as follows: thyroid nodules complicated with Hashimoto's thyroiditis; cystic‐dominated nodules; and calcified nodules, which affect the measurement of the surrounding lesion tissues. Finally, all 281 PTC nodules and 272 NG nodules met the inclusion criteria and were thus included in this study. Then, from June 2019 to August 2019, 200 thyroid nodules (100 PTCs and 100 NGs) that were confirmed by pathology at Tianjin Medical University Cancer Institute and Hospital were included. Fifty thyroid nodules (23 PTCs and 27 NGs) were included in the testing group according to the above inclusion criteria.

### 2.2 Ultrasound examination

Philips iU22 and HD11 ultrasound scanners (California, USA) were used with a probe frequency set to 5‐12 MHz. When the patient was placed in the supine position, the anterior thyroid region was exposed, and the lesion was scanned on the longitudinal, transverse, and oblique planes. The location of the lesion was found, and the sonographic features of the lesion were observed, including its number, size, shape, boundary, length/width ratio, internal echo, peripheral halo, calcification, internal, and peripheral blood supply.

### 2.3 Image analysis method

**Figure 1:**
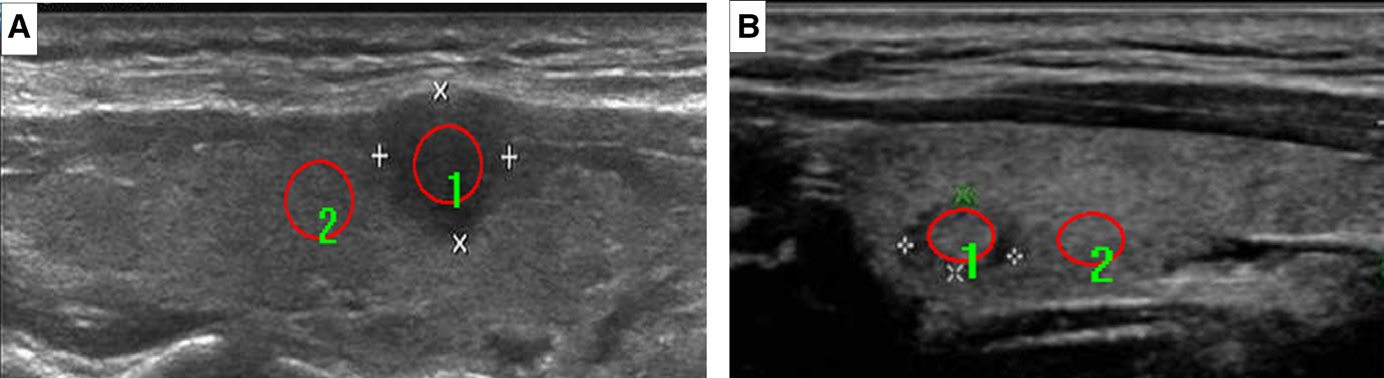
For PTCs with homogeneous echogenicity (A), regions of interest (ROIs) are drawn to include the nodule (1) and the surrounding normal thyroid tissue (2). The mean gray scale values for the PTCs and the surrounding normal thyroid tissue were 44.0 and 70.5, respectively, and the UGSR was 0.625 (44.0/70.5). For NGs with homogeneous echogenicity (B), the regions of interest (ROIs) are drawn to include the nodule (1) and the surrounding normal thyroid tissue (2). The mean gray scale values for the NGs and the surrounding normal thyroid tissue were 70.0 and 84.2, respectively, and the UGSR was 0.878 (70.0/84.2). Note that the ROI (red cycle) should be drawn around the largest area of homogeneous echogenicity nodules. PTCs, papillary thyroid carcinomas; NGs, nodular goiters; UGSR, ultrasound gray scale ratio

**Figure 2:**
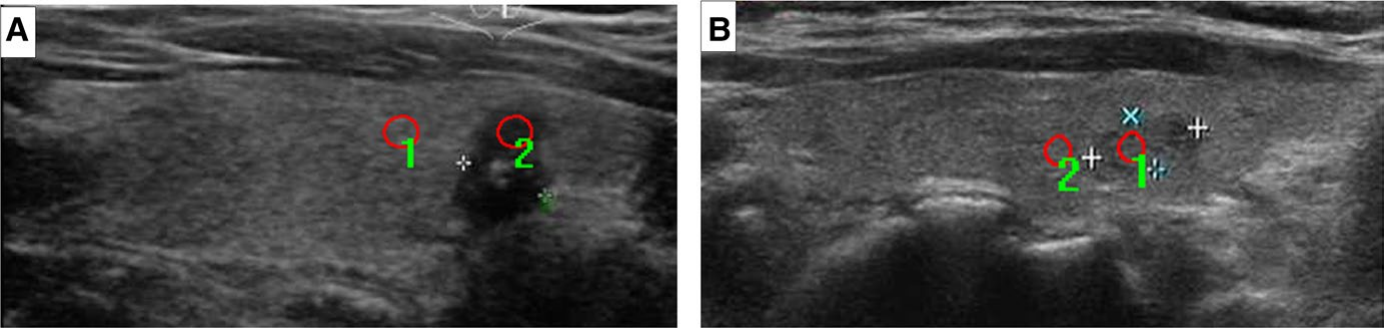
For PTCs with heterogeneous echogenicity (A), regions of interest (ROIs) are drawn to include the nodule (1) and the surrounding normal thyroid tissue (2). The mean gray scale values for the PTCs and the surrounding normal thyroid tissue were 31.9 and 83.8, respectively, and the UGSR was 0.381 (31.9/83.8). For NGs with heterogeneous echogenicity (B), regions of interest (ROIs) are drawn to include the nodule (1) and the surrounding normal thyroid tissue (2). The mean gray scale values for the NGs and the surrounding normal thyroid tissue were 74.7 and 84.9, respectively, and the UGSR was 0.879 (74.7/84.9). Note that the ROI (red cycle) should be drawn around the largest area of the echo‐dominated region of heterogeneous echogenicity nodules. PTCs, papillary thyroid carcinomas; NGs, nodular goiters; UGSR, ultrasound gray scale ratio

Ultrasound images selected from the picture archiving and communication systems (PACS) were analyzed by two radiologists (W. X. and Z. J.), both of whom have 10 years of experience in thyroid lesion ultrasonographic diagnosis. As nodules at the upper and lower poles of the thyroid lack surrounding normal thyroid tissue to compare with during ultrasonic crosscutting scanning, ultrasonic longitudinal images were adopted in this study. Image‐Pro Plus 6.0 (Media Cybernetics) was used to determine the size and measurement area of the PTC nodules, NG nodules, and the surrounding normal thyroid tissue to obtain their gray scale values. The echogenicity intensity was measured in gray scale levels, and their means, minima, maxima, and standard deviations (SDs) were calculated. When measuring the region of interest (ROI) of nodules, the calcified and cystic areas should be avoided. For nodules with homogeneous echoes (Figure 1), the largest ROI should be used. For nodules with heterogenous echoes (Figure 2), the echo‐dominated region and the largest ROI should be used. When measuring the gray scale of the surrounding normal thyroid tissues, its ROI should be consistent with that of the nodule. The UGSR of the PTCs (PTC gray values/gray scale values of the surrounding normal thyroid tissue) and the NGs (NG gray values/gray scale values of the surrounding normal thyroid tissue) was calculated. To evaluate the impact of the size of the nodules on the diagnostic value of the UGSR, we stratified the analysis based on the nodule size (0.3‐1 cm, 1.0 cm‐1.5 cm and 1.5‐2.0 cm, 0.3‐2.0 cm) (Table 1).

**Table 1:** Distribution of different sizes of thyroid nodules in PTCs and NGs

Variants	Thyroid nodule sizes (No.)			
	0.3‐1 cm	1‐1.5 cm	1.5‐2 cm	0.3‐2 cm
PTCs	158	106	17	281
NGs	225	36	11	272
Total	383	142	28	553

Abbreviations: NGs nodular goiters; PTCs, papillary thyroid carcinoma nodules.

### 2.4 Statistical analysis

The Mann‐Whitney U test was applied to compare the two sets of data (PTCs and NGs). SPSS 22.0 (SPSS Inc) was used to draw the ROC curve of the UGSR for distinguishing PTCs and NGs, with the sensitivity as the ordinate and 1‐specificity as the abscissa. On this curve, the closer the point was to the upper‐left corner, the higher the sensitivity and specificity. The point that was the closest to the upper‐left corner represented the best cut‐off value of the sensitivity and specificity. The area under the ROC curve (AUC) was calculated to estimate the overall accuracy of the UGSR. A *P* value less than .05 was considered statistically significant. Finally, the diagnostic performance, including the sensitivity, specificity, positive predictive value (PPV), negative predictive value (NPV), and accuracy of the UGSR, was calculated in the testing group.

## 3 RESULTS

### 3.1 UGSR in distinguishing PTCs and NGs

**Figure 3:**
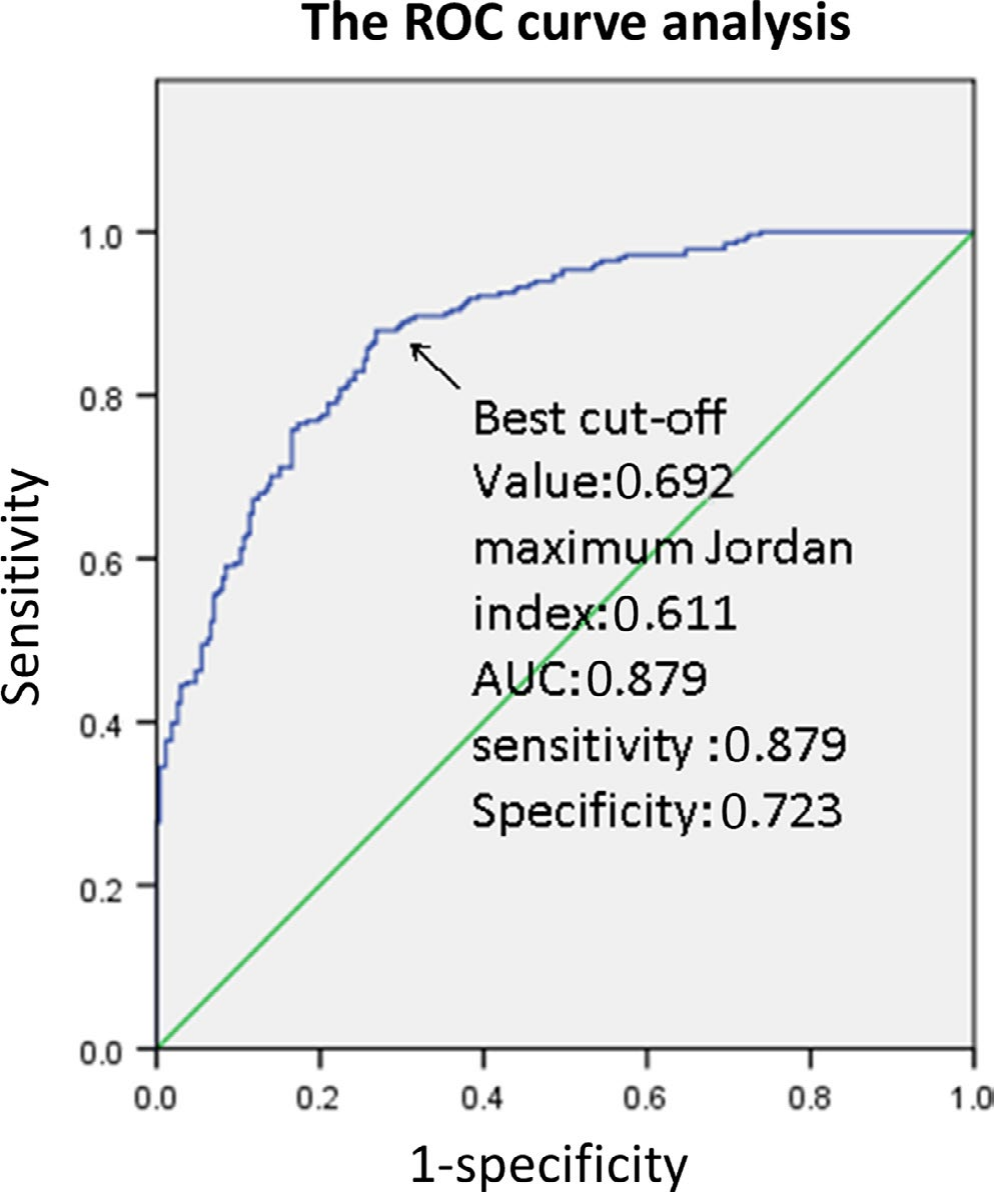
The ROC curve of the UGSR for distinguishing PTCs and NGs in subgroups of different tumor sizes. ROC, receiver operating characteristic; UGSR, ultrasound gray scale ratio; PTCs, papillary thyroid carcinomas; NGs, nodular goiters

For the 281 PTCs and 272 NGs, the range of the UGSRs was 0.211‐0.973 and 0.402‐2.007, respectively. The median UGSRs were 0.520 and 0.829, respectively. The area under the ROC curve (AUC) of the UGSR used for distinguishing PTCs and NGs was 0.879 (95% Cl: 0.851‐0.906), which represented a high diagnostic accuracy. As the UGSR increased, the sensitivity for diagnosing PTCs decreased while the specificity increased. When the maximum Jordan index was 0.611, the best cut‐off value was 0.692, and the corresponding sensitivity and specificity of diagnosing PTCs were 87.9% and 73.2%, respectively (Figure 3).

### 3.2 UGSR in the assessment of different sizes of PTCS

As shown in Table 2, with the increase in the thyroid nodular size (from 0.3 to 2 cm), the sensitivity of diagnosing PTCs decreased while the specificity increased. For the analysis of subgroups of different tumor sizes, when the nodule size was 0.3‐1.0 cm, the best cut‐off value was 0.692 and the corresponding sensitivity and specificity for diagnosing PTCs were 97.5% and 72.4%, respectively; when the nodule size was 1.0‐1.5 cm, the best cut‐off value was 0.629 and the corresponding sensitivity and specificity for diagnosing PTCs were 68.9% and 88.9%, respectively; when the nodule size was 1.5‐2.0 cm, the best cut‐off value was 0.758 and the corresponding sensitivity and specificity in diagnosing PTCs were 58.8% and 90.9%, respectively (Table 2). The AUC of the subgroup of smaller‐sized PTCs (0.3‐1 cm) was higher than that of the subgroup of larger‐sized PTCs (>1 cm) (0.919 vs 0.853 and 0.807 and 0.879, respectively) (Table 2).

**Table 2:** Analysis of ROC curve in the diagnosis of different sizes of thyroid nodules

Sizes	Best cut‐off value	Sensitivity	Specificity	Maximum Jordan index	AUC
0.3‐1.0 cm	0.692	0.975	0.724	0.699	0.919
1.0‐1.5 cm	0.692	0.689	0.889	0.578	0.853
1.5‐2.0 cm	0.758	0.588	0.909	0.497	0.807
0.3‐2.0 cm	0.692	0.879	0.723	0.611	0.879

Abbreviation: AUC, area under the ROC curve.

### 3.3 The diagnostic performance of the UGSR in the testing group

To evaluate the diagnostic performance of the UGSR in the testing group, we compared the ability of the UGSR to distinguish benign and malignant thyroid nodules with pathological diagnoses. The sensitivity, specificity, predictive value (PPV), negative predictive value (NPV), and accuracy of UGSR were 91.3%, 74.1%, 75.0%, 90.9%, and 82.0%, respectively.

## 4 DISCUSSION

The use of ultrasound in clinical practice contributes to the detection of thyroid nodules in medical centers. However, ultrasound also has several drawbacks that lead to uncertainties in the diagnostic process.^13^, ^14^, ^15^ In ultrasound reporting systems, the interobserver agreement reported for echogenicity is quite low.^16^ Additionally, echogenicity is not a significant predictor of malignancy. When describing the internal echogenicity pattern of a thyroid nodule, all the terms that are commonly used to describe the echogenicity are qualitative and subjective, so they cannot provide absolutely objective information about the degree of echogenicity. As illustrated above, the sensitivity and specificity of the degree of hypoechogenicity in the naked‐eye diagnosis of PTCs were 87.2%‐93.8% and 21.8%‐61%.^17^, ^18^, ^19^ Although the sensitivity seemed sufficient, the specificity of the echogenicity in the diagnosis of malignant nodules was still insufficient. The echogenicity intensity of the thyroid nodules quantified by the UGSR was used in our study. When the UGSR was set to the best cut‐off value, the corresponding sensitivity and specificity of diagnosing PTCs were 87.9% and 73.2%, respectively; the specificity was much higher than that in the previous reports.^17^, ^18^, ^19^ Using the UGSR to assess the echo was obviously more objective and accurate than the echogenicity identified by the naked eyes.

Based on previous studies that examined the value of thyroid nodule size as a predictor of malignancy, we advocate the consideration of size discrepancies in distinguishing benign and malignant nodules. Furthermore, in the latest version of the ACR TI‐RADS,^3^ different threshold sizes corresponding to different nodular risk levels were defined for the recommendations of FNA and surgery and prognosis. We hypothesized that different nodule sizes might affect the diagnostic effectiveness of the UGSR. Thus, we divided our analysis into subgroups based on size (0.3‐1 cm, 1.0 cm‐1.5 cm and 1.5‐2.0 cm, 0.3‐2.0 cm). We learned from the results that with the increase in the thyroid nodular size from 0.3 to 2 cm, the sensitivity of the UGSR to diagnose PTCs decreased while the specificity increased. The results of subgroup analysis may be because smaller nodules had higher malignancy rates than larger nodules in the PTC group.^20^, ^21^ Therefore, the predictive value of the hypoechogenicity for malignancy decreased with increasing size. As a quantitative approach to define a nodule's echogenicity, the UGSR's diagnostic value in differentiating malignant and benign thyroid nodules naturally decreased as the nodule size increased. Thus, the nodule size had a considerable impact on the diagnostic value of the UGSR in distinguishing PTCs from NGs. It also indicated the necessity to define the cut‐off values of the UGSR in different subgroups according to the size of nodules. This finding was consistent with the conclusions from other studies in which nodule sizes greatly influenced the diagnostic performance of the thyroid ultrasound due to the lower specificity of smaller thyroid nodules.^9^, ^22^ Appropriate triage by the UGSR in conjunction with size can aid the evaluation of nodules and prediction of malignancy.

Finally, we used a separate testing group to verify the diagnostic performance of the UGSR. We compared the ability of the UGSR to distinguish benign and malignant thyroid nodules with that of the pathological diagnosis. When 0.692 was used as the cut‐off value of the UGSR, the sensitivity and specificity were 91.3% and 74.1%, respectively, which were consistent with the previous results. It was proven that the use of the UGSR was feasible for distinguishing benign and malignant nodules with good diagnostic performance.

Although most thyroid nodules can be differentiated by the morphologic features by conventional gray scale US, the UGSR can handle additional data to overcome the limitations of qualitative and subjective diagnosis. Therefore, it could be a useful objective quantitative measure in distinguishing thyroid nodules. Indeed, a numerical evaluation of the hypoechogenicity (ie, with the UGSR) can precisely quantify the degree of hypoechogenicity and, as a particular feature, could also be included in the new TI‐RADS models. The improvement in the accuracy and specificity with this gray scale analysis could lead to a reduction in unnecessary invasive biopsy procedures and the overdiagnosis or overtreatment of thyroid cancers.

There are several limitations in our study. First, this paper is a retrospective analysis, so selection bias is inevitable, and there may be some differences in the selection and measurement of ROIs for heterogeneous nodules. Second, in this study, we only investigated the value of the UGSR for PTCs and NGs, while the role of the UGSR in other subtypes of thyroid cancers, such as follicular thyroid carcinoma or medullary thyroid carcinoma, needs further study. Third, there are many malignant signs of PTCs, such as microcalcification, solid intranodular blood flow, and taller‐than‐wide shape, while this article only investigated the differentiated diagnostic effect of the UGSR on malignant and benign thyroid tumors. As a result, the value of the UGSR combined with other features in the differential diagnosis of malignant thyroid nodules needs to be studied further. Last but not least, different ultrasound scanners from different companies have their own image quality levels, and different images from different scanners will lead to inconsistent results in the quantitative echogenicity (gray scale) values of the nodules.^23^ In this paper, we measured the gray scale on ultrasound images in only one kind of scanner.

## 5 CONCLUSIONS

The ultrasound gray scale ratio (UGSR) is an objective, numerical estimate of the echogenicity degree in differentiating benign and malignant thyroid lesions and has various diagnostic efficacies in the different sizes of thyroid nodules. Thus, the UGSR can be used as an additional ultrasound parameter in the diagnosis of different‐sized PTCs and NGs. Additionally, we advocate further studies about the actual diagnostic value of the UGSR.

## CONFLICT OF INTERESTS

The authors have no conflict of interest to declare.

## Data Availability

I confirm that my article contains a Data Availability Statement even if no data are available (list of sample statements) unless my article type does not require one.
